# Immersive, Interactive, Intelligent Patient Educational System for Venous Thromboembolism (ChatVTE): Development and Validation Study

**DOI:** 10.2196/82775

**Published:** 2026-04-06

**Authors:** Bin bin Liu, Zhi geng Jin, Zhe qi Zhang, Hong Wang, Hao Wang, Hui Zhang, Chang zhen Li, Fei Qi, Yu tao Guo

**Affiliations:** 1 Chinese People’s Liberation Army Medical School, Chinese People’s Liberation Army General Hospital, Beijing, China; 2 Department of Pulmonary Vascular and Thrombotic Disease, The Sixth Medical Center of Chinese People’s Liberation Army General Hospital, 6 Fucheng Road, Haidian District, Beijing, 100048, China, 86 0-106-695-7703; 3 Department of Cardiology, The Second Medical Center of Chinese People’s Liberation Army General Hospital, Beijing, China; 4 Technical Department, DrBreath Medical Technology Co., Ltd., Beijing, China

**Keywords:** venous thromboembolism, large language model, retrieval-augmented generation, digital health, patient education, psychosocial, patient-centered care

## Abstract

**Background:**

Effective patient education is crucial in preventing venous thromboembolism (VTE), improving patient outcomes, and reducing health care costs. However, traditional educational methods often lack engagement and fail to address individual patient needs comprehensively.

**Objective:**

This study aimed to develop and preliminarily validate an immersive, large language model–based patient education system for VTE designed to promote patient engagement and care adherence by delivering highly relevant, actionable, and patient-centered information.

**Methods:**

We developed ChatVTE, an interactive, intelligent patient education platform, by integrating a retrieval-augmented large language model (Qwen1.5-7B) with text-to-speech and lip-synch technologies. The system’s performance was initially assessed through a comparative evaluation against ChatGPT. This involved using a standardized set of VTE-related questions, administered from December 10 to 31, 2024, with responses rigorously evaluated by 4 VTE domain experts using a 5-point Likert scale for accuracy, completeness, consistency, and safety. Subsequently, we consecutively enrolled a prospective cohort of 25 adult inpatients with VTE from the Departments of Pulmonary Vascular and Thrombotic Diseases and General Surgery at the Sixth Medical Center of the Chinese People’s Liberation Army General Hospital between March 1 and May 31, 2025. These participants engaged with the ChatVTE system throughout their inpatient stay and completed postintervention assessments upon discharge.

**Results:**

Expert evaluation demonstrated that ChatVTE significantly outperformed ChatGPT in accuracy, completeness, consistency (all *P*<.001, *r*>0.5), and safety (*P*=.01, *r*=0.327). Among the 25 enrolled patients (age: mean 55.4, SD 13.2 years), ChatVTE achieved high average scores (mean score >4.0/5.0) in 8 of the 9 experience dimensions evaluated but received a notably lower score in the emotional support domain (1.92/5.0).

**Conclusions:**

This study validates the feasibility of ChatVTE in the management of patients with VTE, demonstrating its potential to enhance the quality of patient–health care provider interaction and the efficacy of knowledge dissemination. These preliminary findings suggest that ChatVTE could be a valuable tool for improving patient education and facilitating shared clinical decision-making.

## Introduction

Venous thromboembolism (VTE), encompassing deep venous thrombosis and pulmonary embolism, is a predominant cause of avoidable mortality and morbidity on a global scale, ranking among the top 3 cardiovascular emergencies in high-income nations [1]. Despite progress in prophylactic strategies, a significant gap remains in patient education, which is a fundamental aspect of sustained VTE management [2]. Current evidence-based interventions largely concentrate on the optimization of anticoagulant therapy. However, recent research emphasizes the critical importance of education in improving adherence to prophylaxis, recognizing symptoms, and reducing the risk of recurrent hospitalizations [3-5]. Patients exhibit a significant interest in acquiring knowledge about VTE through various educational modalities [6]. Nonetheless, conventional educational approaches, which typically involve clinician-led verbal instruction and static written materials, are limited by their one-way nature and lack of continuity.

Mobile health technologies, such as smartphone apps, have emerged as a promising means for delivering dynamic and personalized educational content [7]. Nevertheless, many current mobile health platforms use rule-based content delivery systems, which fail to address patients’ evolving needs. Large language models (LLMs), such as GPT-4, can generate contextual responses, offering a promising avenue for personalized patient education [8-10]. However, this potential is constrained by the tendency of general-domain models to produce inaccurate or outdated medical information. Retrieval-augmented generation (RAG) architectures address these limitations by grounding responses in real-time access to authoritative medical databases and clinical guidelines [11], a method validated in prior clinical applications [12,13].

Building upon recent technological advancements, we previously developed and validated a mobile venous thromboembolism app (mVTEA), a mobile app for VTE management. Its core functionalities, including risk stratification, medication adherence monitoring, and teleconsultation services, have been shown to enhance adherence to prophylaxis [14]. However, user feedback identified significant limitations in educational interactivity and personalized engagement. To address these challenges, we designed ChatVTE, an immersive intelligent system integrating LLMs with mVTEA’s existing framework. This system uses RAG architecture to dynamically synthesize evidence-based responses from curated clinical guidelines and patient education repositories, enabling real-time, context-aware interactions addressing complex clinical inquiries. This study aimed to develop and preliminarily validate an immersive LLM-driven patient education system for VTE.

## Methods

### Study Design and Setting

This study was designed as a pilot study to evaluate the feasibility and user experience of ChatVTE in a real-world clinical setting. It was conducted using a multiphase approach: an initial technical validation phase involving expert evaluation, followed by a prospective single-arm cohort study assessing patient experience. Both phases were conducted in a tertiary medical center in Beijing, China, specifically involving patients from the Departments of Pulmonary Vascular and Thrombotic Diseases and General Surgery at the Sixth Medical Center of the Chinese People’s Liberation Army General Hospital. This study adhered to the iCHECK-DH (Guidelines for the Reporting on Digital Health Implementations; Checklist 1) [15].

### ChatVTE Platform Engineering

#### Overview

**Figure 1.**
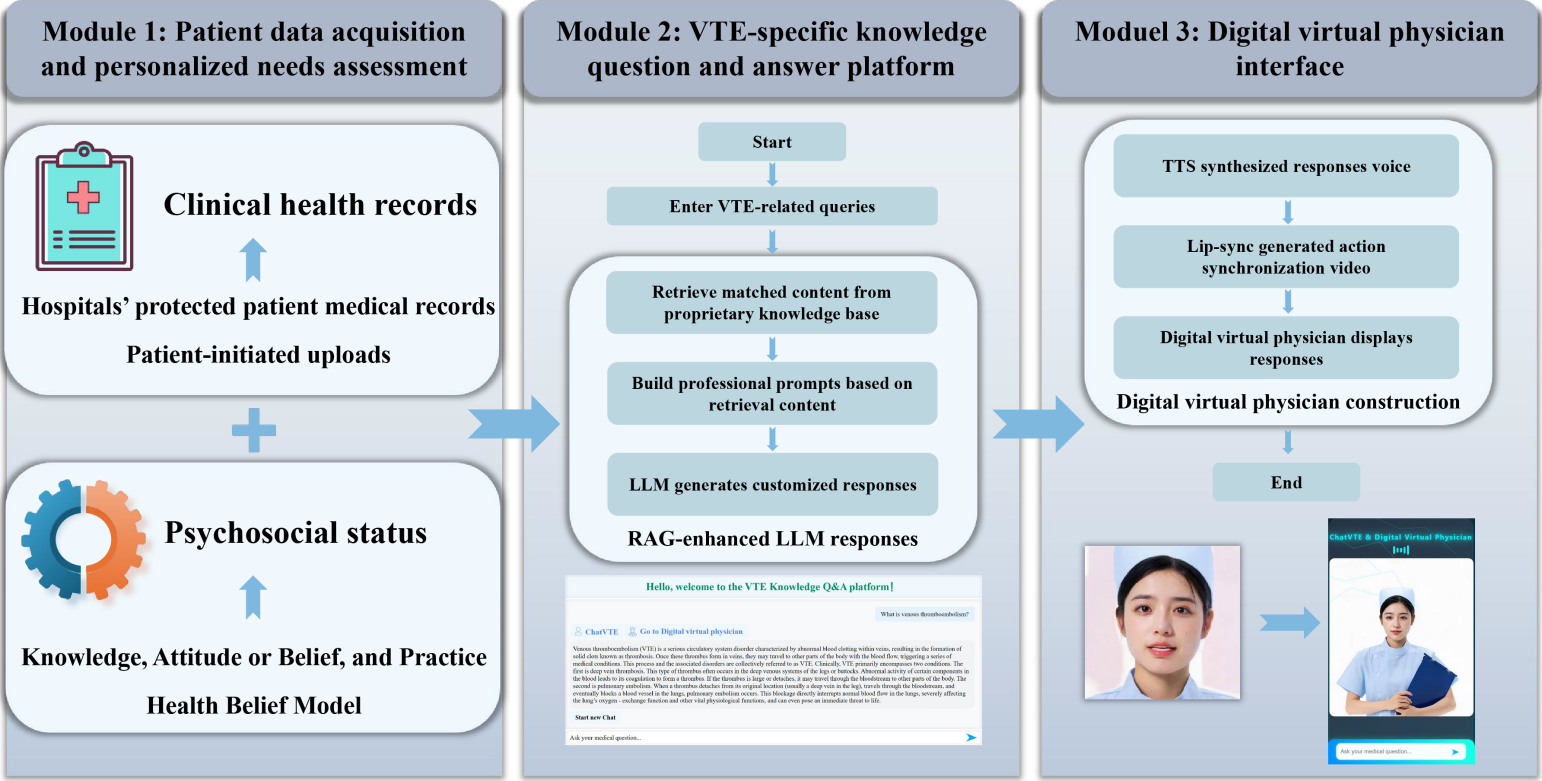
ChatVTE architecture: an immersive retrieval-augmented generation (RAG) large language model (LLM) patient education platform for venous thromboembolism (VTE) integrating data capture, personalized needs assessment, a knowledge question and answer engine, and a virtual clinician. TTS: text-to-speech.

ChatVTE is an interactive, intelligent patient education platform for VTE, developed by integrating a retrieval-augmented LLM (Qwen1.5-7B), text-to-speech (TTS) synthesis, and lip-synch technologies. This platform accepts VTE-related queries, retrieves validated content from a dedicated knowledge repository, processes the information via RAG technology, and then delivers a tailored response. The development process systematically constructed 3 interconnected core modules (Figure 1). Through this integration, the platform provides patients with personalized VTE education content, self-management strategies, and guidance on health-promoting behaviors. The core purpose is to deliver highly relevant, actionable, and patient-centered information, thereby promoting patient engagement and adherence to care.

#### Module 1: Patient Data Acquisition and Personalized Needs Assessment Module

This module supports comprehensive patient profiling by securely integrating data from clinical records and patient-reported questionnaires. Clinical data were digitized from patient-authorized clinical documents using an optical character recognition (OCR)–based extraction process and organized into structured clinical entities to support personalized patient education and needs assessment [16]. Additionally, the module deploys periodic, structured questionnaires via the mVTEA platform to evaluate patients’ knowledge, attitude, and practice (KAP) profiles as well as health belief model (HBM) domains pertinent to VTE. Questionnaire responses are securely consolidated and analyzed to quantify health-literacy levels, delineate behavioral patterns, and detect knowledge gaps or adherence barriers. Integrating these psychosocial metrics with the clinically derived data enables precise characterization of each patient’s needs and informs the generation of tailored educational material and intervention strategies within ChatVTE.

#### Module 2: VTE-Specific Knowledge Question and Answer Platform

This module serves as the central intelligence for delivering evidence-based VTE information. A comprehensive knowledge repository was meticulously curated from authoritative open-access internet resources, including professional society guidelines (eg, from the European Society of Cardiology and the American Heart Association), peer-reviewed literature, and established biomedical databases such as PubMed. The aggregated material encompassed the full spectrum of VTE topics, including etiology, risk factors, clinical presentation, diagnostic techniques, pharmacologic and nonpharmacologic management, prevention, and long-term care strategies. All retrieved data were subjected to a stringent preprocessing workflow that eliminated obsolete entries, harmonized medical terminology, and verified factual accuracy. The refined corpus was partitioned into discrete knowledge units and converted into vector representations. These embeddings enable a RAG engine to rapidly retrieve contextually relevant content for the Qwen1.5-7B LLM. The model subsequently integrates the extracted evidence to produce precise, context-sensitive, and patient-oriented textual responses. To prevent misinterpretation of system outputs as clinical orders, ChatVTE was explicitly designed as a patient education system rather than a clinical decision or order-entry tool. The interface includes clear disclaimers, and the system does not generate executable clinical instructions, thereby reducing the risk of misinterpretation as medical orders.

#### Module 3: Digital Virtual Physician Interface

**Figure 2.**
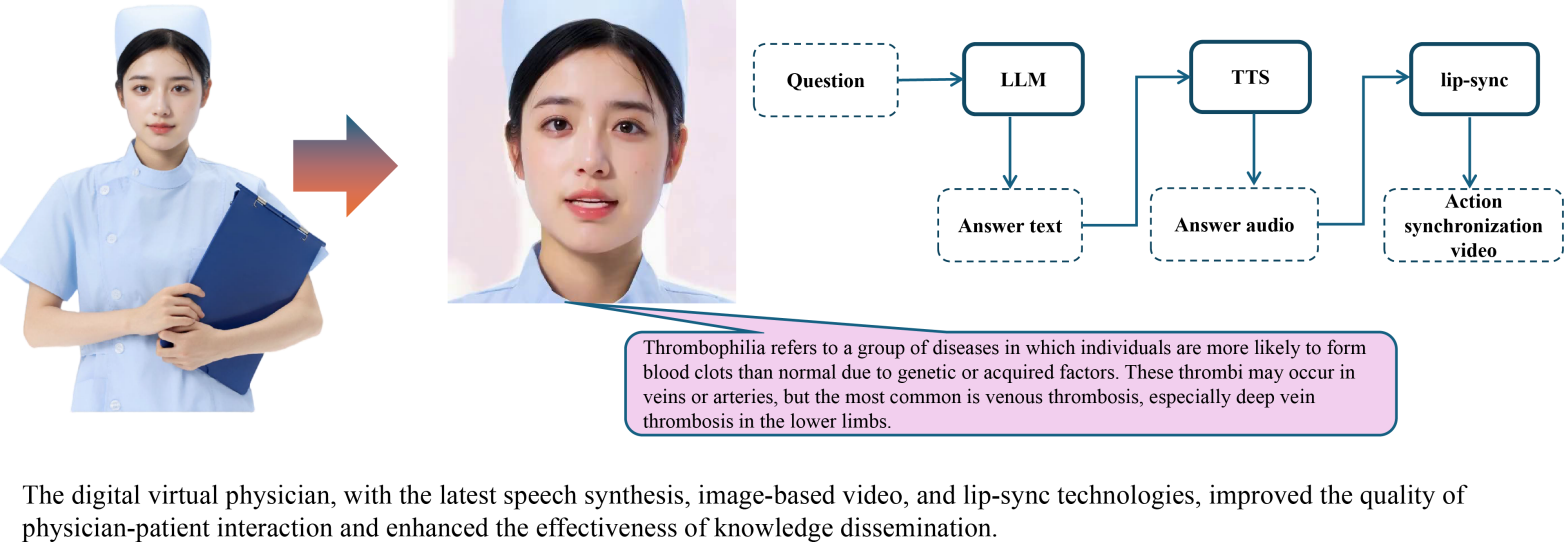
Digital virtual physician: patient education. LLM: large language model; TTS: text-to-speech.

This module facilitates an immersive and naturalistic communication experience. It transforms the textual output from the VTE-specific knowledge question and answer platform (module 2) into professional medical-style streaming audio via a specialized TTS module (Figure 2). The TTS module uses a pretrained medical voice corpus to ensure clear articulation of complex terminology, appropriate intonation for empathy and authority, and precise pronunciation. Concurrently, lip-synch technology generates precisely synchronized lip movements and microexpressions for the digital virtual clinician. This system maps speech segments to visual animations in real time, achieving a rendering rate of 24 to 30 frames per second with asynchronous latency of under 50 milliseconds. This process overlays the animations onto the virtual clinician’s facial model, significantly improving the visual realism and engagement of the interactive presentation. For example, we posed a query “What is venous thromboembolism?” to ChatVTE. The resulting streaming video is provided in Multimedia Appendix 1.

### Evaluation Protocol

A dual-phase evaluation protocol was implemented to assess the system’s technical output quality and its practical utility for patients.

#### Expert Comparative Validation

We rigorously benchmarked the performance of ChatVTE against ChatGPT (GPT-4) between December 10 and 31, 2024. GPT-4 was selected as the high-performance general-purpose LLM baseline to assess the added value conferred by ChatVTE’s domain-specific optimization. We accessed GPT-4 via its official web interface ChatGPT, with web search disabled to ensure responses derived solely from its internal knowledge. Default safety and content moderation settings were applied without modification. This configuration was designed to simulate a typical scenario in which an end user directly interacts with a general-purpose LLM to seek medical information, thereby establishing a clear baseline for evaluating the added value of ChatVTE’s specialized design.

Both models responded to a standardized set of 30 VTE-related questions, which were carefully selected from the mVTEA education section, and comprehensively spanned key management domains (Table S1 in Multimedia Appendix 2). These included pathophysiology; risk factors; diagnosis and clinical classification; acute and long-term treatment strategies; care for special populations (cancer, pregnancy, etc), and essential patient education for self-management and follow-up. Each of the 30 questions was posed to both ChatVTE and ChatGPT on 3 separate occasions: an initial round and 2 repeat rounds at 72-hour intervals, using semantically equivalent but differently worded formulations. Thus, a total of 180 individual responses were generated (2 models×30 questions×3 generations). The initial round of responses was used for evaluating accuracy, completeness, and safety; all 3 rounds were considered for the consistency assessment. Responses were independently assessed by 4 VTE domain experts (each possessing ≥7 years of clinical experience), who were blinded to the model source (Textbox 1). Responses were rated using 5-point Likert scales for accuracy, completeness, and consistency (1: very inaccurate, incomplete, or inconsistent; 5: very accurate, complete, or consistent). Safety, defined as the potential risk posed by erroneous or misleading information, was assessed on a separate 5-point severity scale (0: no risk; 4: extreme risk). Detailed anchor definitions are provided in Table S2 in Multimedia Appendix 2.

Textbox 1. **Summary of assessment area, measurement tool, and primary outcome for ChatVTE evaluation.**

**Expert-comparative performance vs ChatGPT (these items were scored in a blinded review on a 1-5 Likert scale, with a higher score indicating a better evaluation)**

- accuracy
- completeness
- consistency
- safety (this item was rated on a separate 0-4 Likert scale, with a lower score indicating greater safety)

**Patient-overall experience (these items were scored on a 1-5 Likert scale, with a higher score indicating a better evaluation)**

- acceptability
- convenience
- timeliness
- fluency
- comprehensibility
- accuracy
- empathy
- satisfaction
- recommendability

#### Patient Experience Assessment

From March 1 to May 31, 2025, we consecutively enrolled 25 adult inpatients with a confirmed VTE diagnosis into a single-arm, single-center pilot study. Participants were provided with access to and guidance on using the ChatVTE system throughout their hospitalization. Upon discharge, each individual completed a structured 9-item questionnaire, expanded from earlier mVTEA evaluations, to preliminarily assess their overall experience (Table S3 in Multimedia Appendix 2). The questionnaire measured 9 dimensions: acceptability, convenience, timeliness, fluency, comprehensibility, accuracy, empathy, satisfaction, and recommendability, using a 5-point Likert scale (higher scores indicate more favorable evaluations; Textbox 1).

To ensure the instrument’s suitability for this study’s context, we used an iterative development and qualitative validation process. Prior to formal data collection, a qualitative pilot test was conducted with a small group of hospitalized patients with VTE (n=5). The primary objective was to evaluate the instrument’s face validity and content validity, specifically focusing on the clarity of terminology, ease of comprehension, and respondent burden. During this pilot phase, we conducted brief cognitive debriefings to identify any ambiguous phrasing. On the basis of patient feedback, we linguistically refined several items to ensure that they were culturally and clinically appropriate for the target population. While formal psychometric analysis (eg, Cronbach α) was not performed at this stage due to the small sample size of the pilot group, this iterative process ensured that the questionnaire was content-valid and optimized for the clinical study.

### Ethical Considerations

This study involved human participants and was approved by the ethics committees of the Sixth Medical Center of the Chinese People’s Liberation Army General Hospital (HZKY-PJ-2022-21). Written informed consent was obtained from all participants before their inclusion in the study. Participants were informed of the purpose of the study, the nature of their interaction with the ChatVTE, and their right to withdraw at any time without consequences. No compensation was provided for participation. Clinical documents and inpatient records were acquired through secure, hospital-approved channels, including patient-authorized document upload and OCR-based extraction [16]. OCR processing was performed within a controlled hospital environment. Direct personal identifiers were separated from clinical content immediately after extraction, and only deidentified limited datasets were used for downstream processing. Identifiable information was retained locally and was not transmitted to or processed by the language model. Access to patient data was restricted to authorized users through role-based authentication, and data access and processing events were logged to support auditability. All data were stored on secure in-country servers in accordance with institutional and national data protection regulations.

To protect participant privacy and data confidentiality, ChatVTE did not support real-time, bidirectional integration with the hospital information system during this pilot phase.

### Statistical Analysis

Previous methodological work has demonstrated that pilot studies are appropriately sized to detect feasibility or implementation-related problems rather than to achieve statistical significance. Using a problem-detection framework, a sample size in the range of approximately 20 to 30 participants is sufficient to identify, with 95% confidence, feasibility or workflow-related issues that occur with a probability of at least 10% to 15% among study participants [17]. Given that the primary objectives of this study were to evaluate system feasibility and user experience in a relatively homogeneous patient population, a target sample size of 25 participants was considered adequate to meet these exploratory aims and to inform the design of subsequent larger-scale studies.

Quantitative data are summarized descriptively. Continuous variables are presented as mean (SD) or median (IQR). Expert evaluation scores for each model were calculated as the average ratings per question. Given that the same 30 questions were evaluated for both ChatVTE and ChatGPT, between-model comparisons were performed using the paired Wilcoxon signed-rank test. Effect sizes were quantified using the *r* statistic, calculated as $\text{r = }\text{Z}\text{/}\sqrt{\text{N}}$, where Z is the test statistic from the Wilcoxon signed-rank test. On the basis of the Cohen convention, an *r* value of approximately 0.1 indicates a small effect, approximately 0.3 indicates a medium effect, and *r*≥0.5 indicates a large effect [18,19]. The sign of Z (and thus *r*) indicates the direction of the difference: a positive value indicates that ChatVTE received higher scores than ChatGPT, and a negative value indicates the opposite. The absolute value of *r* reflects the magnitude of the effect. Patient evaluation scores were determined by the average score for each question. A 2-sided *P*<.05 was considered statistically significant. All statistical analyses were performed using SPSS Statistics (version 26.0; IBM Corp) and R (version 4.4.1; R Foundation for Statistical Computing).

## Results

### Expert Comparative Validation Findings

**Figure 3.**
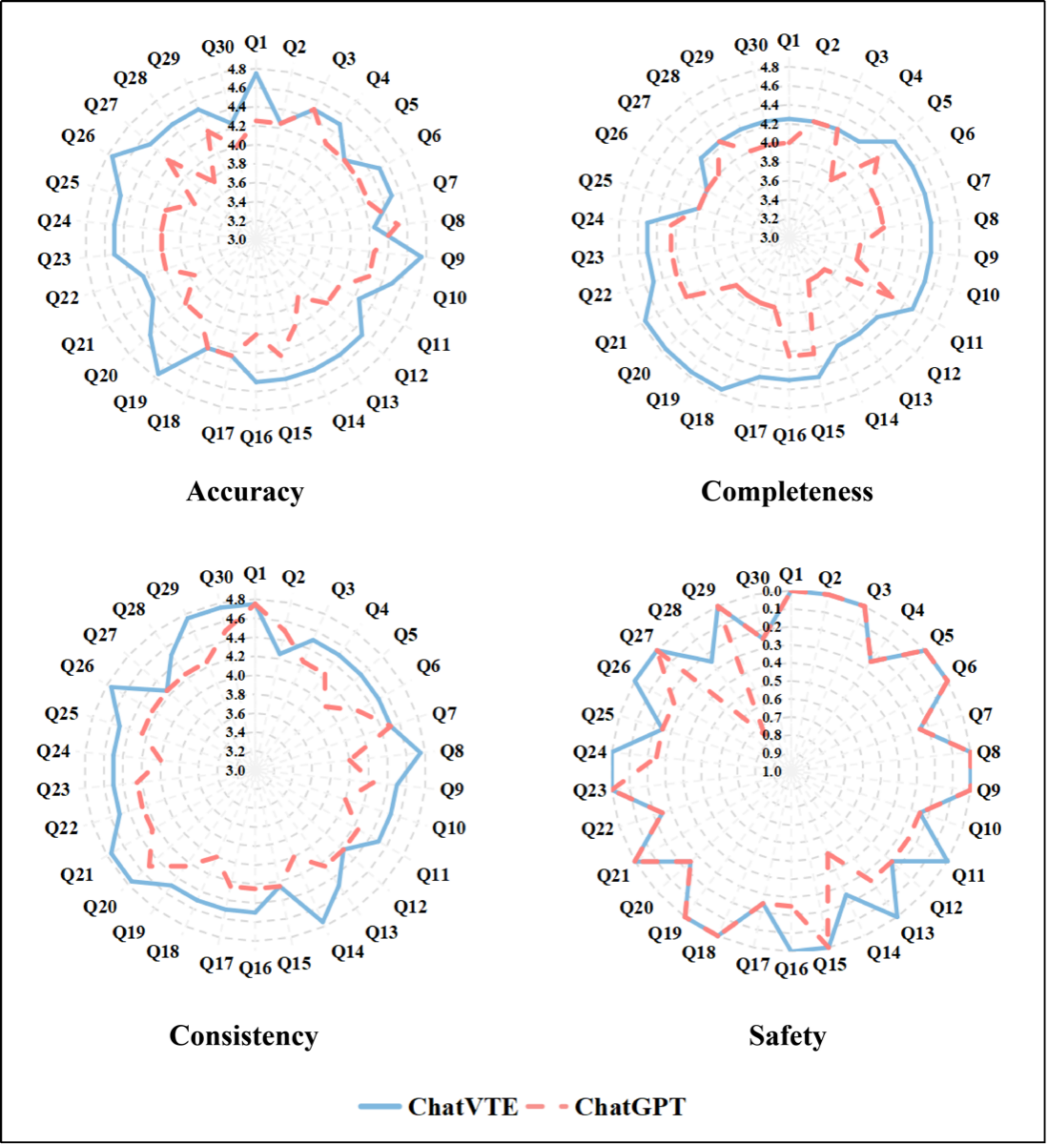
Comparison between ChatVTE and ChatGPT for validity evaluation. Scores for accuracy, completeness, and consistency are rated on a scale of 1 to 5, with higher scores indicating better performance. For safety, scores range from 0 to 4, with lower scores reflecting lower risk and thus higher safety. Q: question.

Four VTE experts independently appraised all outputs, and the aggregated ratings are presented in Figure 3. Across the full question set, ChatVTE achieved significantly higher scores than ChatGPT in both accuracy (mean 4.46, SD 0.16 vs mean 4.11, SD 0.20; Z=4.287; *r*=0.553; *P*<.001) and completeness (mean 4.41, SD 0.20 vs mean 3.98, SD 0.25; Z=4.429; *r*=0.572; *P*<.001). In the analysis restricted to the 8 items on postdischarge management, ChatVTE again significantly outperformed the comparator for accuracy (mean 4.50, SD 0.13 vs mean 4.00, SD 0.19; Z=2.546; *r*=0.637; *P*=.01) and completeness (mean 4.25, SD 0.19 vs mean 4.09, SD 0.13; Z=2.546; *r*=0.559; *P*=.03). The consistency rating was also higher for ChatVTE (mean 4.53, SD 0.16) than for ChatGPT (mean 4.25, SD 0.17; Z=4.289; *r*=0.554; *P*<.001). Regarding perceived safety—defined as the likelihood of potentially harmful misinformation—ChatVTE received a lower score compared to ChatGPT (mean 0.09, SD 0.12 vs mean 0.16, SD 0.18; Z=−2.530; *r*=0.327; *P*=.01), indicating a safer profile for ChatVTE. For instance, in response to the specific prompt about managing missed anticoagulant doses (question 28), ChatGPT received its highest risk rating (score=2, denoting moderate concern). In contrast, only 1 reviewer assigned ChatVTE a score of 1, indicating minimal concern. The 4 independent raters showed strong concordance, with their ratings falling within a 1-point range for 86.7% (26/30) or more of the items across all domains and models. No item exhibited a rating discrepancy exceeding 2 points on the scale.

### Patient Experience Assessment

**Figure 4.**
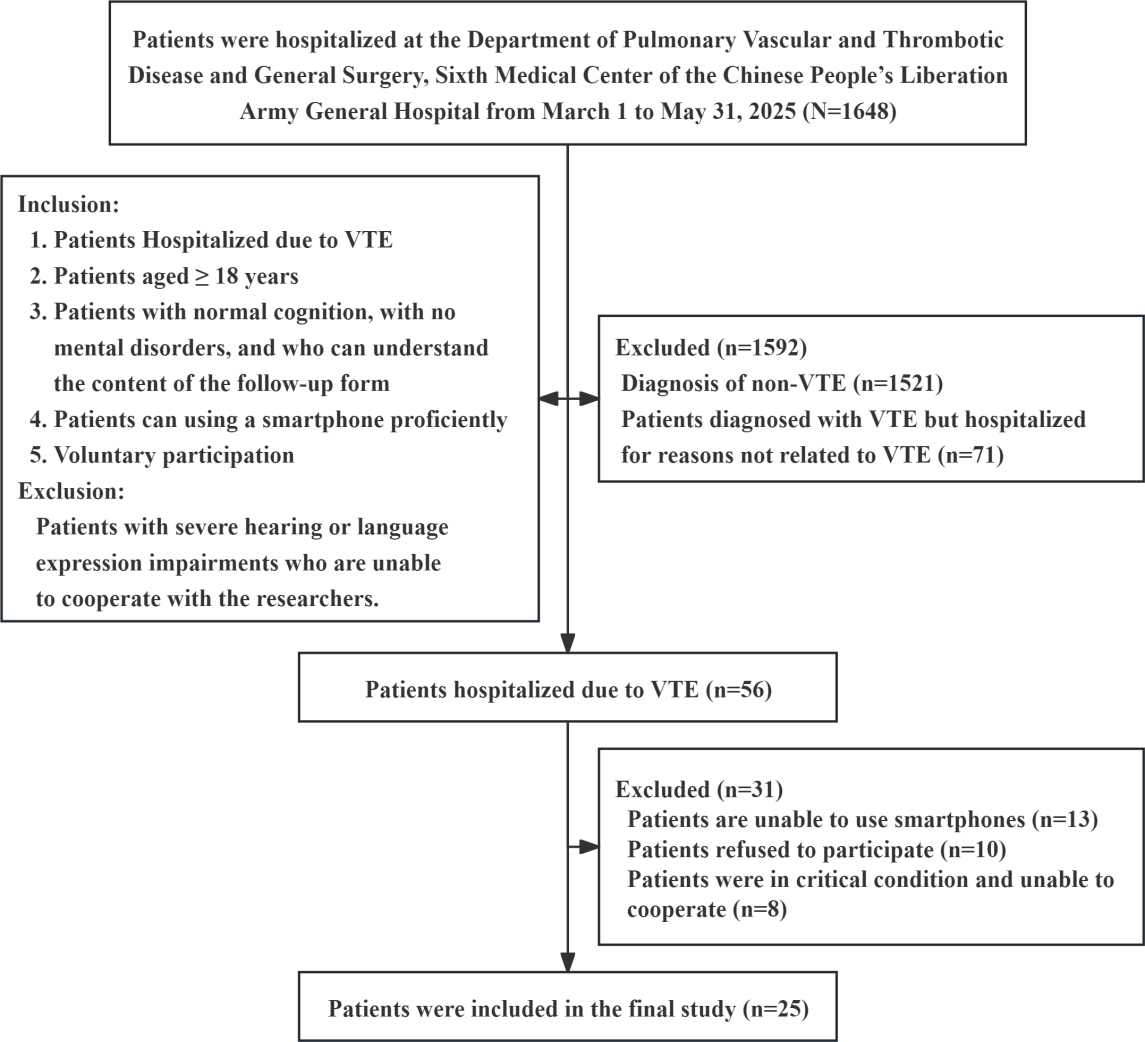
Flowchart for evaluating the use of ChatVTE in patients with venous thromboembolism (VTE).

**Figure 5.**
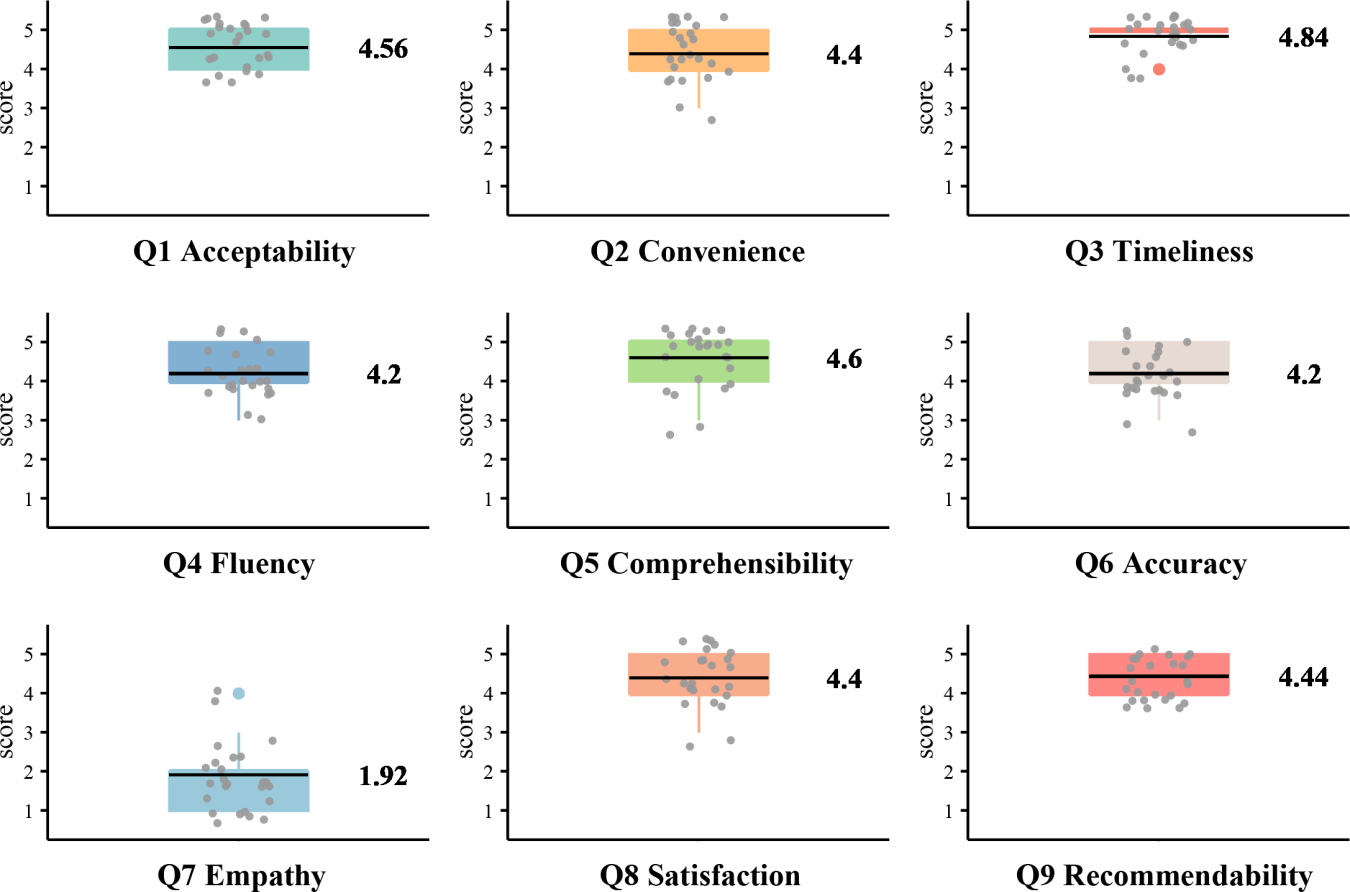
Evaluation results for patients with venous thromboembolism after using ChatVTE in various dimensions (higher scores mean better evaluations). Q; question.

All 25 participants returned valid questionnaires (Figure 4). Baseline demographic and clinical characteristics are summarized in Table 1. The average duration of ChatVTE use was 7.6 (SD 3.9; IQR 5.5‐9.5) days. Figure 5 shows that the highest mean rating was for response timeliness (item 3: 4.84/5), while the lowest was for emotional support (item 7, 1.92/5). Overall satisfaction and willingness to recommend the platform to other patients with VTE received mean scores above 4.4 on the 5-point scale, indicating generally favorable user acceptance.

**Table 1.** Baseline characteristics of all patients (N=25).

Characteristics	Patients
Age (years), mean (SD)	55.4 (13.2)
Age group (years), n (%)	
≥60	11 (44)
<60	14 (56)
Female, n (%)	10 (40)
Education level, n (%)	
More than high school	16 (64)
Less than high school	9 (36)
BMI (kg/m^2^), mean (SD)	25.5 (3.5)
≥28 kg/m^2^^a^, n (%)	6 (24)
Comorbidities, n (%)	19 (76)
Cancer	8 (32)
Diabetes	5 (20)
Hypertension	4 (16)
Rheumatic immune disease	3 (12)
ChatVTE use time (days), mean (SD)	7.6 (3.9)

a BMI ≥28 kg/m2 is defined as obesity according to the Chinese guidelines.

## Discussion

### Principal Findings

In this study, we introduced ChatVTE, an LLM-based system specialized for VTE care. It innovatively integrates patient health information, personalized patient needs, RAG-augmented LLM, and a digital virtual physician to form a convenient and comprehensive patient education and support platform. The dual-stage evaluation demonstrated ChatVTE’s strong performance in expert and patient assessments.

ChatVTE aims to facilitate patient-centered VTE care, which requires precise management based on personal health information. The system uses an LLM-enhanced multimodal OCR with patient-uploaded data, enabling continuous tracking of disease progression. Beyond acute treatment, poor self-management engagement and low behavioral adherence remain critical barriers to effective long-term VTE care. Evidence suggests that interventions based on KAP and HBM can improve patients’ self-management, and integrating patients’ values enables LLMs to generate content and recommendations that are tailored to patients’ preferences [20-23]. To address these barriers, we incorporated the evaluation of patients’ KAP levels and HBM status into ChatVTE. By combining patients’ personal health information with their psychosocial characteristics, ChatVTE can deliver customized VTE educational resources, self-management strategies, and health-promoting behaviors, thereby promoting patient-centered care.

RAG addresses inherent shortcomings of general-purpose LLMs, such as hallucination, and its effectiveness is well-established [11-13,24-26]. ChatVTE leverages a RAG-based LLM, which is significantly superior to a general-purpose LLM (ChatGPT). The high accuracy and completeness of the model’s response demonstrated its feasibility in the care of patients with VTE. In expert assessments, no responses received a risk score ≥2 (moderate or higher). This preliminary finding suggests that the system may contribute to reducing the risk of adverse outcomes caused by misguidance, offering a promising tool for patient education and care. Higher response consistency indicated that it can provide more stable and reliable guidance for patients with VTE, reducing clinical decision-making confusion and patient compliance risks caused by conflicting information. On the basis of a structured questionnaire survey among patients hospitalized for VTE, ChatVTE proved overall highly satisfactory (average score ≥4.2/5), although there remained room for improvement in empathy. Furthermore, ChatVTE used TTS and lip-synch technology to convert generated text into streaming video, thereby creating an interactive digital virtual VTE physician. This significantly enriches the patient’s experience and is particularly user-friendly for patients with limited digital health literacy (such as older individuals and those with limited education).

### Comparison With Prior Work

Consistent with prior studies, ChatVTE further demonstrates the feasibility of applying a RAG-augmented LLM in specific diseases (VTE) [13,27,28]. Most general-purpose and medical-specific LLMs are text-based, which is relatively monotonous and provides a poor user experience for older patients and those with limited education. An LLM-driven, 3D, hyperrealistic interactive intelligent digital human system named MetaTutor has changed this paradigm [29]. It can interact with users through voice and text and simulate facial expressions and body movements based on the content of the conversation. ChatVTE created an interactive digital virtual VTE physician that was the first digital virtual physician driven by specialty-specific VTE knowledge retrieval LLM. It provides patients with an immersive communication experience, capable of explaining complex medical information and providing accurate responses to queries. As ChatVTE was primarily designed for patient self-service, its interactive capabilities within virtual scenarios are currently less advanced than those of MetaTutor. However, the use of virtual scenarios will be a key area for future optimization of ChatVTE. Regarding response consistency, Kelly et al [30] designed an augmented LLM for patients with type 2 diabetes mellitus. Their evaluation method involved repeatedly inputting identical questions, which does not account for the natural variation in how patients phrase queries in real-world settings. This approach may not fully reflect the model’s adaptability to synonymous and heterogeneous queries. Textual matching based on cosine similarity makes it impossible to judge the consistency of core information transmission in terms of clinical significance. However, this may be related to their original intention of designing the model to improve the health literacy of patients with type 2 diabetes mellitus through robust content output.

ChatVTE’s limited empathetic capacity stems from its fundamental lack of genuine emotional experience. Its responses are generated solely by imitating language patterns in training data, which hampers its ability to truly comprehend and contextualize patients’ complex emotional states and underlying needs [31]. While optimized for delivering accurate clinical information, ChatVTE’s prompts and response templates may insufficiently prioritize the recognition and validation of patient emotions. For instance, the system might correctly answer a factual question about anticoagulation but fail to acknowledge the accompanying anxiety expressed by the patient. However, careful design of LLM prompts (limiting response scope, setting goals and formats, standardizing interaction rules, etc) can improve the empathy of model output results [30,32]. Future versions will integrate emotion-aware prompting to acknowledge patient concerns, incorporate a library of validating response templates (eg, for anticoagulation-related anxiety), and implement a simple keyword-triggered rule to recommend human consultation upon detecting signals of severe distress. These modifications offer practical strategies for mitigating the common issue of insufficient empathy in medical LLMs.

Privacy and security concerns surrounding personal health information make integration into LLMs challenging. Ge et al [33] developed an LLM for liver-related diseases based on a platform that meets protected health information standards, providing new strategies for achieving patient-centered care. Developed for the care and education of patients with VTE, ChatVTE enables the extraction of pertinent health information from protected hospital medical records under strict controls, while also allowing patients to voluntarily upload their clinical information. In addition, the integration of social and psychological status evaluation into patient care has been absent from previous similar studies. The practical application of disease-specific LLM is also important. For instance, NeuroBot, an LLM for neurosurgical patient education, was evaluated using focus groups comprising health care professionals [28]. This subjective method and the exclusive focus on professionals may limit the generalizability of the findings to the target patient population. In contrast, the LLM developed by Adhikary et al [34] for menstrual health education was evaluated more comprehensively among both health care professionals and diverse volunteers.

### Future Research Directions

ChatVTE, based on the mVTEA, provides patients with precise and personalized VTE information in a convenient manner. This helps reduce reliance on nonprofessional online content, enhances patients’ awareness of disease risks, and improves adherence to preventive measures. Although it cannot replace clinical consultations, it has the potential to optimize communication between physicians and patients. Future research should consider the following aspects. First, ChatVTE requires further optimization. This includes enhancing its empathetic responses through tailored prompt words and integrating it with the hospital information system. Second, future studies should integrate ChatVTE into the entire process of patient management and evaluate its effectiveness in patient education and achieving patient-centered care in clinical practice, as well as its effects on reducing adverse events and improving long-term prognosis in patients with VTE.

### Limitations

This study has several limitations. First, as a single-arm, single-center pilot study with a modest sample size (N=25), the patient experience assessment of ChatVTE limits the generalizability of the findings to other clinical settings and broader patients with VTE. The limited sample size may result in insufficient statistical power to detect anything other than large effects. Consequently, the positive user ratings should be interpreted cautiously as preliminary evidence of acceptability. The lack of a control group in this pilot study means that positive user feedback could be influenced, in part, by the increased attention inherent to participation in a study (eg, the Hawthorne effect) [35]. This is an inherent limitation of a single-arm feasibility study.

Another limitation is the patient experience questionnaire used in this study. While it was refined through a qualitative pilot process to ensure face validity and clinical relevance, it has not yet undergone formal large-scale psychometric validation. Therefore, the findings regarding patient experience should be considered preliminary evidence of user acceptance, and a comprehensive, multicenter evaluation of the ChatVTE system should be conducted in the future.

In addition, the comparison between ChatVTE and ChatGPT, while offering referential value, is contextually limited. ChatGPT represents a general-purpose LLM without medical-domain optimization, whereas ChatVTE is a proprietary LLM designed for education and management of patients with VTE. Consequently, the observed advantages of ChatVTE should be interpreted primarily as validation of its customized architecture in achieving specific clinical objectives, rather than as a direct, head-to-head comparison of model capabilities. Future evaluations should incorporate medical-specialized LLMs, particularly those focused on VTE, to provide a more comprehensive and equitable assessment. Although the preliminary safety assessment of ChatVTE was encouraging based on expert ratings of a limited question set, real-world deployment must address practical issues such as digital literacy gaps, user trust, data privacy, and ethical compliance. If patients interpret ChatVTE-generated recommendations as definitive medical advice, it could lead to potential harm. Therefore, it is necessary to establish safeguards such as human monitoring and legal disclaimers.

### Conclusions

ChatVTE, an augmented LLM-based platform, synthesizes individual clinical information and psychosocial characteristics to enable novel strategies for patient education and patient-centered care. It provides patients with an immersive experience through an interactive artificial intelligence conversational agent. These preliminary findings suggest that ChatVTE may serve as a promising, scalable tool for supporting patient-centered VTE care. Future research should rigorously assess its effectiveness on clinical outcomes, long-term usability, and implementation within diverse health care settings.

## Supplementary material

10.2196/82775 Multimedia Appendix 1 Response from ChatVTE to the question “what is venous thromboembolism?”

10.2196/82775 Multimedia Appendix 2 The venous thromboembolism–related issues used in the ChatVTE validation process, the corresponding expert scoring criteria, and the structured questionnaire for the patient experience survey.

10.2196/82775 Checklist 1 iCHECK-DH checklist.

## Abbreviations

HBM: health belief model
KAP: knowledge, attitude, and practice
LLM: large language model
mVTEA: mobile venous thromboembolism app
OCR: optical character recognition
RAG: retrieval-augmented generation
TTS: text-to-speech
VTE: venous thromboembolism
