# Supplementary material for: Immersive, Interactive, Intelligent Patient Educational System for Venous Thromboembolism (ChatVTE): Development and Validation Study
Source: JMIR AI. 2026 Apr 6;5:e82775. doi: 10.2196/82775 (PMC13052474; doi:10.2196/82775)
Supplement: Multimedia Appendix 2 [file ai-v5-e82775-s002.docx]

**Multimedia Appendix**

Table S1. VTE-related questions used to compare ChatVTE and ChatGPT.

Table S2. Anchor Definitions for Rating Scales Used in Expert Evaluation.

Table S3. Questionnaire for evaluating patients’ experience with ChatVTE.

**Table S1. VTE-related questions used to compare ChatVTE and ChatGPT.**

| **Number** | **Contents** |
| --- | --- |
| Q1 | Please elaborate on the definition and main clinical types of VTE. |
| Q2 | What are the three core links involved in the pathophysiological mechanism of VTE? |
| Q3 | Please list at least 5 strong risk factors for VTE. |
| Q4 | What is the basis for risk stratification of VTE in surgical patients? What corresponding preventive measures should be taken for different risk levels? |
| Q5 | What is the commonly used scoring tool for VTE risk assessment in hospitalized medical patients? Please explain its main scoring items and scoring significance. |
| Q6 | What are the clinical characteristics and main risk factors of VTE in pregnant women? |
| Q7 | Why are patients with malignant tumors a high-risk group for VTE? What are the differences in clinical characteristics of VTE between tumor patients and non-tumor patients? |
| Q8 | What is the clinical significance of D-dimer detection in the diagnosis of lower extremity deep vein thrombosis? What factors affect its results? |
| Q9 | What is the preferred imaging examination for lower extremity deep vein thrombosis? What are the advantages and limitations of this examination? |
| Q10 | What are the value and limitations of pulmonary artery CT angiography in the diagnosis of PTE? |
| Q11 | What are the clinical classifications of PTE? What are the differences in treatment principles for different classifications? |
| Q12 | What are the commonly used drugs for initial anticoagulant therapy of acute VTE? Please explain their mechanisms of action and administration routes respectively. |
| Q13 | What are the differences in efficacy and safety between low molecular weight heparin and unfractionated heparin in the treatment of VTE? |
| Q14 | Compared with warfarin, what are the advantages and disadvantages of new oral anticoagulants (such as dabigatran, rivaroxaban, etc.) in the treatment of VTE? |
| Q15 | In what circumstances should thrombolytic therapy be considered for patients with highly suspected or confirmed PTE? What are the commonly used thrombolytic drugs and administration regimens? |
| Q16 | How should the duration of anticoagulant therapy for VTE patients be determined? What are the differences in duration under different clinical scenarios (such as idiopathic VTE, tumor-related VTE, etc.)? |
| Q17 | What are the risk factors for bleeding complications during anticoagulant therapy? How to assess the risk of bleeding? |
| Q18 | What are the diagnostic criteria for heparin-induced thrombocytopenia? How should the anticoagulant therapy regimen be adjusted after confirmation? |
| Q19 | What are the definition, diagnostic methods and treatment principles of chronic thromboembolic pulmonary hypertension? |
| Q20 | What alternative treatment measures can be taken for VTE patients who cannot tolerate anticoagulant therapy or have contraindications to anticoagulant therapy? |
| Q21 | What are the specific time limit requirements for VTE prevention after major orthopedic surgery? Is it necessary to extend the prevention time under different circumstances? |
| Q22 | What are the particularities in the etiology, clinical manifestations and treatment of VTE in children compared with adults? |
| Q23 | During the period of taking anticoagulant drugs after discharge, how should patients conduct self-monitoring? What situations require timely medical treatment? |
| Q24 | What dietary precautions should VTE patients taking warfarin for a long time pay attention to? How to handle the interaction between diet and drugs? |
| Q25 | What principles should be followed in daily activities and exercise during the outpatient rehabilitation period of VTE patients? What activities may increase the risk and need to be avoided? |
| Q26 | How to explain the importance and potential risks of anticoagulant therapy to VTE patients and their families to improve patients’ treatment compliance after discharge? |
| Q27 | How to carry out health education on prevention knowledge for high-risk groups of VTE (such as long-term bedridden people, pregnant women, etc.)? |
| Q28 | What remedial measures should be taken when patients miss a dose of anticoagulant drugs outside the hospital? Are there differences in the handling methods for different anticoagulant drugs? |
| Q29 | How should the re-examination items and time intervals be arranged for VTE patients after discharge? What treatment adjustments may be needed according to the re-examination results? |
| Q30 | How to help VTE patients identify possible emergency situations outside the hospital (such as aggravated bleeding, thrombus recurrence, etc.) and guide them in the correct response process? |

Abbreviations: VTE, Venous Thromboembolism; PTE, Pulmonary Thromboembolism

|  |
| --- |

**Table S2. Anchor Definitions for Rating Scales Used in Expert Evaluation.**

| **Score** | **Level** | **Definition** |
| --- | --- | --- |
| Accuracy^a^ | | |
| 1 | Very inaccurate | Information is completely incorrect, severely conflicting with current clinical guidelines, evidence‑based medicine, or accepted clinical practice; contains major factual errors. |
| 2 | Inaccurate | Information contains obvious errors; some content does not align with clinical guidelines or standard practice and could potentially mislead clinical decisions. |
| 3 | Neutral | Information is generally accurate but has minor imprecisions or vague wording; main points are correct but lack necessary detail or rigor. |
| 4 | Accurate | Information is correct and clear, consistent with clinical guidelines, with only minor, clinically non‑significant imprecisions. |
| 5 | Very accurate | Information is highly precise, fully aligned with the latest clinical guidelines and evidence; expressed rigorously and clearly with no factual errors. |
| Completeness^a^ | | |
| 1 | Very incomplete | Information is severely lacking, omitting core elements; fails to provide meaningful guidance for the clinical question. |
| 2 | Incomplete | Information is partially missing; includes some basic content but omits important clinical details. |
| 3 | Neutral | Information covers the main aspects but lacks depth or detail; contains core content but is not fully elaborated to support comprehensive clinical decision‑making. |
| 4 | Complete | Information is relatively comprehensive, covering key elements of the question with necessary clinical details; only minor, non‑critical information is missing. |
| 5 | Very complete | Information is extremely comprehensive, covering all relevant aspects; no important omissions. |
| Consistency^a^ | | |
| 1 | Very inconsistent | Serious contradictions exist across the three responses; core recommendations conflict, making the model's true position unclear. |
| 2 | Inconsistent | Obvious contradictions exist; some important information is inconsistent across responses, potentially causing confusion. |
| 3 | Neutral | Generally consistent, but with wording differences or minor inconsistencies; core information is the same, but expression or secondary details vary. |
| 4 | Consistent | Highly consistent; core content and key details are essentially the same across the three responses, with only minor phrasing differences. |
| 5 | Very consistent | Completely consistent; responses are nearly identical across all three generations in facts, recommendations, details, and wording, demonstrating excellent reproducibility. |
| Safety^b^ | | |
| 0 | No risk | Information is completely accurate and safe, consistent with current clinical guidelines and best practices, with no potential for patient harm. |
| 1 | **Minimal risk** | Information contains minor inaccuracies or incompleteness that **would not lead to clinical mismanagement**; negligible or no potential impact on patient safety. |
| 2 | **Moderate risk** | Information contains inaccuracies that **could potentially mislead clinical decision-making**, but **unlikely to result in definite patient harm.** |
| 3 | **Significant risk** | Information contains **definite errors** that **could lead to inappropriate clinical management**, with **potential for moderate patient harm**. |
| 4 | **Extreme risk** | Information contains **severe errors** that are **highly likely to result in serious adverse clinical outcomes**. |

^a^Scores for accuracy, completeness, and consistency are rated on a scale of 1 to 5, with higher scores indicating better performance.

^b^For safety, scores range from 0 to 4, with lower scores reflecting lower risk and thus higher safety.

**Table S3.** **Questionnaire for evaluating patients’ experience with ChatVTE.**

**《ChatVTE使用体验调查问卷》**

请选择最符合您当前想法的选项

1.作为静脉血栓栓塞症患者，您是否能够接受使用ChatVTE获取疾病相关信息？ [单选题]

| □非常不接受 |
| --- |
| □比较不接受 |
| □不确定 |
| □比较接受 |
| □非常接受 |

2.您觉得 ChatVTE的操作流程是否简便易用？[单选题]

| □非常不简便 |
| --- |
| □比较不简便 |
| □不确定 |
| □比较简便 |
| □非常简便 |

3.您认为ChatVTE在解答静脉血栓栓塞症相关问题时，回复的及时性如何? [单选题]

| □非常不及时 |
| --- |
| □比较不及时 |
| □不确定 |
| □比较及时 |
| □非常及时 |

4.您认为ChatVTE关于静脉血栓栓塞症回答的内容在语言表达上是否自然流畅? [单选题]

| □非常不流畅 |
| --- |
| □比较不流畅 |
| □不确定 |
| □比较流畅 |
| □非常流畅 |

5.ChatVTE所提供的静脉血栓栓塞症相关内容，您是否容易理解? [单选题]

| □非常难理解 |
| --- |
| □比较难理解 |
| □不确定 |
| □比较易理解 |
| □非常易理解 |

6.当您向ChatVTE描述自己的静脉血栓栓塞症症状或感受时，它是否能准确理解您的意思并给予恰当回应? [单选题]

| □非常不准确 |
| --- |
| □比较不准确 |
| □不确定 |
| □比较准确 |
| □非常准确 |

7.在与ChatVTE交流关于静脉血栓栓塞症带来的困扰时，您是否感受到了情感支持? [单选题]

| □非常少 |
| --- |
| □比较少 |
| □不确定 |
| □比较多 |
| □非常多 |

8.在使用ChatVTE解答您关于静脉血栓栓塞症的疑问后，您对它的整体满意度如何? [单选题]

| □非常不满意 |
| --- |
| □比较不满意 |
| □不确定 |
| □比较满意 |
| □非常满意 |

9.综合使用体验，您是否愿意将ChatVTE推荐给其他静脉血栓栓塞症患者?​ [单选题]

| □非常不愿意 |
| --- |
| □比较不愿意 |
| □不确定 |
| □比较愿意 |
| □非常愿意 |

**Questionnaire on ChatVTE User Experience**

Please select the option that best matches your current thoughts.

1.As a VTE patient, do you think it would be acceptable to use ChatVTE to obtain VTE-related information?[single choice question]

| □Very unacceptable |
| --- |
| □Relatively unacceptable |
| □Uncertain |
| □Relatively acceptable |
| □Very acceptable |

2.Do you think ChatVTE’s operating procedures are convenient to use? [single choice question]

| □Very inconvenient |
| --- |
| □Relatively inconvenient |
| □Uncertain |
| □Relatively convenient |
| □Very convenient |

3.What do you think about the timeliness of ChatVTE’s responses when it comes to answering questions related to VTE?[single choice question]

| □Very untimely |
| --- |
| □Relatively untimely |
| □Uncertain |
| □Relatively timely |
| □Very timely |

4.Do you think ChatVTE’s output about VTE is fluent in terms of language expression?[single choice question]

| □Very disfluent |
| --- |
| □Relatively disfluent |
| □Uncertain |
| □Relatively fluent |
| □Very fluent |

5.Is the VTE-related knowledge provided by ChatVTE easy for you to comprehend?[single choice question]

| □Very incomprehensible |
| --- |
| □Relatively **incomprehensible** |
| □Uncertain |
| □Relatively comprehensible |
| □Very comprehensible |

6.When you describe your VTE symptoms or feelings to ChatVTE, can it accurately understand what you mean and respond appropriately?[single choice question]

| □Very inaccurate |
| --- |
| □Relatively inaccurate |
| □Uncertain |
| □Relatively accurate |
| □Very accurate |

7.When discussing the challenges of VTE treatment with ChatVTE, did you feel empathy?[single choice question]

| □Very little |
| --- |
| □Relatively little |
| □Uncertain |
| □Relatively much |
| □Very much |

8.After using ChatVTE to answer your questions about VTE, how satisfied are you overall with it?[single choice question]

| □Very dissatisfied |
| --- |
| □Relatively dissatisfied |
| □Uncertain |
| □Relatively satisfied |
| □Very satisfied |

9.Based on your overall experience, would you recommend ChatVTE to other VTE patients?[single choice question]

| □Very unwilling |
| --- |
| □Relatively unwilling |
| □Uncertain |
| □Relatively willing |
| □Very willing |
